# Opportunities and Challenges for Drug Development: Public–Private Partnerships, Adaptive Designs and Big Data

**DOI:** 10.3389/fphar.2016.00461

**Published:** 2016-12-06

**Authors:** Oktay Yildirim, Matthias Gottwald, Peter Schüler, Martin C. Michel

**Affiliations:** ^1^Institute of Pharmacology, University Duisburg-EssenEssen, Germany; ^2^Department of Drug Discovery, Bayer Pharma AGBerlin, Germany; ^3^Department of Drug Development Services CNS, ICON Clinical ResearchLangen, Germany; ^4^Department of Pharmacology, Johannes Gutenberg UniversityMainz, Germany

**Keywords:** drug development, public–private partnership, investigator-initiated studies, adaptive trial design, big data, informed consent, privacy

## Abstract

Drug development faces the double challenge of increasing costs and increasing pressure on pricing. To avoid that lack of perceived commercial perspective will leave existing medical needs unmet, pharmaceutical companies and many other stakeholders are discussing ways to improve the efficiency of drug Research and Development. Based on an international symposium organized by the Medical School of the University of Duisburg-Essen (Germany) and held in January 2016, we discuss the opportunities and challenges of three specific areas, i.e., public–private partnerships, adaptive designs and big data. Public–private partnerships come in many different forms with regard to scope, duration and type and number of participants. They range from project-specific collaborations to strategic alliances to large multi-party consortia. Each of them offers specific opportunities and faces distinct challenges. Among types of collaboration, investigator-initiated studies are becoming increasingly popular but have legal, ethical, and financial implications. Adaptive trial designs are also increasingly discussed. However, adaptive should not be used as euphemism for the repurposing of a failed trial; rather it requires carefully planning and specification before a trial starts. Adaptive licensing can be a counter-part of adaptive trial design. The use of Big Data is another opportunity to leverage existing information into knowledge useable for drug discovery and development. Respecting limitations of informed consent and privacy is a key challenge in the use of Big Data. Speakers and participants at the symposium were convinced that appropriate use of the above new options may indeed help to increase the efficiency of future drug development.

## Background

In 2002 about 50% of all prescriptions in the U.S. were filled with generics; this has increased to 88% in 2014 (Munos, 2016). Nevertheless, generics accounted for only 17% of total drug expenditure in 2014. In other words, the historic activity of the pharmaceutical industry has provided physicians and patients with a treasure trove of medications which provide adequate treatment for many conditions at a rather low price. This historic success of the pharmaceutical industry has developed into a challenge for its future existence. For diseases with existing treatments, novel treatments must provide improved efficacy and/or tolerability to a major extent; minor improvements are no longer seen as innovation and hence are not reimbursed at branded prices. On the other hand, successful treatments are still lacking for many diseases but this has a reason. Either they have proven difficult to treat, for instance schizophrenia or progression of Alzheimer’s disease, or they are rare or otherwise of unclear commercial value as, for instance, antibiotics.

**FIGURE 1:**
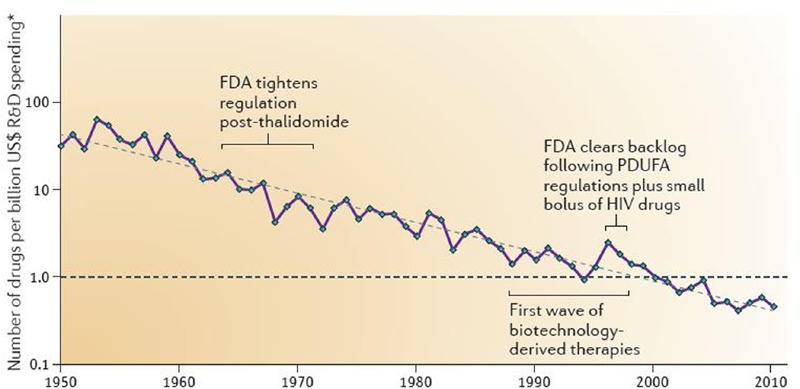
**Number of new drugs approved by the U.S.** Food and Drug Administration per inflation-adjusted billion of US $ spent on Research and Development. Reproduced with permission from Scannell et al. (2012).

Drug development cost has steadily been soaring since the 1950s; actually, Research and Development (R&D) costs per newly approved drug has linearly increased over time – on a logarithmic scale with a doubling of costs approximately every 9 years (Scannell et al., 2012) (**Figure 1**). Accordingly, in contrast to often quoted 1.6 billion US $ for developing a single drug, it has been estimated that costs per drug brought to the market in 1997–2011 is 4 billion US $ or more with a range of 3.7 billion incurred by Amgen (33.2 billion expenses for R&D with a total of nine new drugs) to 11.8 billion incurred by Astra Zeneca (59.0 billion for R&D with a total of five new drugs) (Herper, 2012). The difference between the two estimates is largely driven by attrition, i.e., the inclusion of costs for drugs that failed in development. Industry has reduced attrition due to aspects of pharmacokinetics and bioavailability but has been less successful with other reasons of attrition such as drug efficacy; commercial, toxicology, and clinical safety reasons for attrition may even increase (Kola and Landis, 2004). Despite these improvements, late-stage attrition rates remain at an estimated 75% (Grainger, 2015) and, hence, is a major cost-driver in drug development. The societal demand for truly innovative drugs, i.e., those addressing major unmet medical needs, is likely to worsen this trend as “high innovation” is inherently associated with “high risk” and, therefore, increases the probability of attrition.

The combination of increasing costs of drug development and largely capped budgets for medication challenges the current business model of the pharmaceutical industry. Therefore, new models for a more effective and less costly drug development are needed. Some reported models focus on use of non-clinical or translational data to improve predictions of safety (Bowes et al., 2012) or efficacy (Dolgos et al., 2016). Others focus on target-centric approaches, particularly for new biological entities (Swinney and Anthony, 2011), more rapid paths to clinical proof-of-concept (Owens et al., 2015), or attempts to identify candidates for early termination, i.e., before the most expensive late-stage clinical development starts (Peck et al., 2015), or more systematic portfolio review to systematically challenge development candidates (Cook et al., 2014). Of note, risk mitigation is not only an important topic for the pharmaceutical industry but also in academic drug discovery (Dahlin et al., 2015). Even radical models, such as letting patients/subjects pay for their participation in clinical trials (Emanuel et al., 2015) have been proposed. Which, if any, of these new models will lead to major improvements in the productivity of drug discovery and development is a matter of debate. However, the fact that many different approaches are tested shows that none of them has proven to be the path of choice until now. Although more new medicines were approved in 2015 by the US Food and Drug Administration (Mullard, 2016a) or the European Medicines Agency (European Medicines Agency, 2016; Mullard, 2016b) than in several previous decades, the pressure between increasing development costs per launched drug and largely capped societal expense remains to challenge the existing business model of major pharmaceutical companies.

Two possible avenues are frequently seen as promising opportunities for a more cost-effective drug discovery and development. One of them is co-operative models, which can represent co-operation between pharmaceutical companies or public–private-partnerships (PPP). As stated by Elias Zerhouni, “we must acknowledge that no single institution, company, university, country, or government has a monopoly on innovation” (Zerhouni, 2014). Another is the emergence of big data, be it derived from genomics or from accumulating health-related data from various sources. Against this background, experts from academia, industry, regulatory authorities and patient organizations gathered in Berlin (Germany) on 29.-30.1.2016 to discuss the impact and potential of co-operative models and big data on the future of drug development. This was the 4th annual symposium within a series entitled “Pharmaceutical Medicine,” which was launched in 2013 by the Medical School of the University of Duisburg Essen.

## Types of Public–Private-Partnerships

Partnerships help participants to do things neither could do alone or to do them in a more cost-effective manner, as highlighted by Theo Meert (Janssen, Beerse, Belgium). Thus, complex challenges can hardly ever be addressed by a single academic or commercial entity; partnerships allow pooling of expertise, knowledge and resources and may lead to cross-fertilization. This becomes obvious when it is considered that global annual academic and commercial Research and Development (R&D) expenditure in the life sciences is about 250 billion €, whereas even large pharmaceutical companies invest only a couple of billion, i.e., a low single digit percentage of the global investment (Anonymous, 2015). Engagement in partnerships may help companies to leverage their own investment against that of the global community, albeit with the possible consequence that revenue may also need to be shared.

**FIGURE 2:**
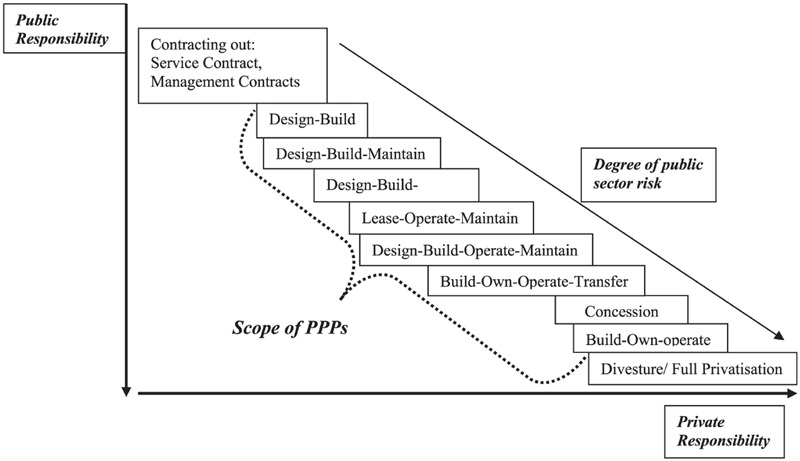
**Scale and scope of private and public responsibility in Public–Private-Partnerships.** Reproduced with permission from Roehrich et al. (2014).

Partnerships can be classified based on the types of participants or on scope and duration of the project, which may fall in the competitive or pre-competitive space. Each of them comes with a specific balance of responsibilities between the public and the private partner (**Figure 2**). At the participant level, partnerships can involve collaboration between companies. For instance, representing collaboration in the competitive space, two companies may work together for the development of companion diagnostics of precision medicines (Fridlyand et al., 2013). Partnerships can also involve multiple pharmaceutical companies, typically for pre-competitive aspects of drug discovery and development. The TransCelerate initiative in the US is an example of this (Gill, 2014).

More frequently, partnerships involve pharmaceutical companies and public institutions, i.e., PPP. PPP not only assist the pharmaceutical industry in improving its productivity; they can also help to fund and sustain the development of academic drug discovery capabilities that benefit society by improving the value gained from publicly funded research (Tralau-Stewart et al., 2009). The almost exponential growth of publications on PPP between 1990 and 2010 testifies to their increasing role (Roehrich et al., 2014). Moreover, not only academic institutions but also foundations increasingly engage in PPP (de Vrueh et al., 2014). PPP can also operate in the competitive or the pre-competitive space. Examples of pre-competitive PPP co-funded by multiple companies, governments, academic institutions and foundations are listed in **Table 1**. Other approaches include corporate mini-labs at academic institutions such as the Mitsubishi Genetic Therapies Centre at Imperial College, sponsored research such as GSK’s academic Alternative Discovery Initiative at Imperial College, or proof-of-concept funds such as Johnson & Johnson collaboration with Imperial College (Tralau-Stewart et al., 2009).

**Table 1:** Examples of pre-competitive public–private partnerships.

Name	Purpose	Region	Website
Scottish Translational Medicine Research Collaboration	Training in translational medicine and therapeutics	Scotland	http://stmti.mvm.ed.ac.uk
Innovative Medicines Initiative	Strengthening of drug research and development in Europe	European Union	www.imi.europa.eu
Dundee Kinase Consortium	Protein kinase research and screening	Global	www.kinase-screen.mrc.ac.uk
Structural Genomics Consortium	Drug discovery related to under-studied areas of human genome	Global	http://www.thesgc.org
SNP Consortium	Gene polymorphism research	Global	http://internationalgenome.org
Biomarker Consortium	Biomarker identification and validation	Global	www.biomarkersconsortium.org

Partnerships can also be classified based on scope and intended duration, for instance project-based collaborations between a company and a specific group of investigators, strategic alliances between a company and an entire academic institutions or large, multi-partner consortia such as those under the umbrella of the Innovative Medicines Initiative (IMI) of the European Union and the European Pharma Association EFPIA. The benefits and challenges in the various types of partnerships were discussed based on specific examples. Martin C. Michel (Johannes Gutenberg University, Mainz, Germany) reported on a case study, in which project-based collaborations with various academic investigators were used in the development of a first-in-class molecule, the β_3_-adrenoceptor agonist mirabegron, now approved for the treatment of the overactive bladder syndrome (Michel and Korstanje, 2016). In this case, project-based collaborations with leading academic groups were used to address evolving mechanistic understanding of the condition to be treated, development and validation of tools (antibodies and radioligands) to detect the drug target at the protein level, impact of gene polymorphisms of the drug target and questions of desensitization. This specific program also fostered relationships with leading academic groups in the field and, thereby, helped to build acceptance for a new treatment modality. Key challenge in these collaborations were the alignment of timelines in academic research with those of commercial drug development and resolving legal issues in setting up contracts between the partners. In a recent survey of academic investigators engaging in project-based, non-clinical collaboration with the pharmaceutical industry, complex contract negotiations were seen as a major hurdle for collaboration, interestingly equally being attributed to legal departments of companies and academic institutions (Amiri and Michel, 2015). Other case studies of project-based collaborations in drug development have recently been reported (Modjtahedi et al., 2014; Michel et al., 2015). Academic investigators engaged in non-clinical drug-related research expressed strong interest in such collaborations (Amiri and Michel, 2015). In a systematic analysis of non-clinical publications presumably reflecting work performed concomitant with commercial drug development, 75% of all such publications had a corresponding author from academia (Köster et al., 2016b). Thus, project-based collaborations between pharmaceutical companies and individual academic groups are an important component of translational drug development, but the specific needs and approaches may vary considerably between projects, even within a company (Köster et al., 2016a).

Strategic alliances between a single pharmaceutical company and a single academic institution are another type of PPP. This option was discussed by Ruth Wellenreuther [German Cancer Research Center (DKFZ), Heidelberg, Germany] and Holger Hess-Stump (Bayer, Berlin, Germany) based on the alliance between their institutions (Wellenreuther et al., 2012). The DKFZ is a mainly government funded organization founded in 1964 with a strong track record in cancer research, as exemplified by two of their researchers, Harald zur Hausen and Stefan Hell, being awarded Nobel Prizes in Physiology or Medicine in 2008 and 2014, respectively. The partnership between DKFZ and Bayer is characterized by several features: Firstly, it is non-exclusive. This means that both partners maintain a range of other strategic partnerships. For instance, DKFZ has such partnerships locally with academic institutions such as the Heidelberg University Medical Center, nationally the German Cancer Consortium, and internationally the Karolinska Institute in Sweden and the National Cancer Institute in the US, and commercial organizations such as IBM, Roche and Siemens Healthcare. Bayer in turn also has a broad spectrum of alliances with academic and commercial partners, for instance in oncology including the MD Anderson Cancer Center, Cancer Research UK, Takeda and Amgen. Second, strategic alliances are long-term oriented, risk and reward sharing agreements between partners with complementary expertise and the shared goal to develop new treatment options. They require significant contributions from both partners in terms of content, resources and funding. This combination allows pursuing highly complex and innovative projects that single partners could not have handled on their own. The alliance between DKFZ and Bayer HealthCare started in 2009. Complementary expertise contributed by the partners include a deep knowledge on the molecular mechanisms of cancer, innovative target ideas, expertise in novel emerging research areas, novel mechanistic assays and clinical expertise and ability to perform clinical studies on the side of DKFZ; on the Bayer side, it includes expertise in drug discovery and development, target validation strategies, established technology platforms for target validation, assay development for high-throughput screening, compound library and medicinal chemistry, preclinical drug development, regulatory approval process and marketing and sales (Wellenreuther et al., 2012; Thong, 2016). As part of the risk and reward sharing approach, the partners share funding of joint projects for a total of so far 6 million Euro per year (currently about 15 projects active). Since April 2013 a joint lab for cancer immunotherapy is operative. Moreover, there is a range of joint activities including organization of scientific conferences, joint project teams, seminars and visits. Bayer has a licensing option for the joint project results to develop projects into commercially successful products, in which case the DKFZ will participate in financial revenues. Both partners realize that alliances may fail. Analysis of failing alliances in general has shown that this is most often due to poor relationships between the partners, which includes lack of understanding for cultural differences, poor trust, poor communication and poor conflict resolution. The second most frequent cause of alliance failure is poor legal and financial terms. In contrast, poor strategy and business planning account for only a minor fraction of alliance failures. As a conclusion, the DKFZ/Bayer alliance has realized the strategic importance of professional alliance management. This includes buy-in from all management levels, high strategic fit and complementary expertise, involvement of alliance managers from negotiation phase until end of cooperation, awareness of the cultural differences and drivers, clearly defined governance structures and processes for interaction, dedicated resources from both sides and regular communication (Lessel and Douglas, 2010). In the DKFZ/Bayer alliance, there is a Joint Research Review Committee, which recommends projects, and Joint Steering Committee, which decides on budget. Both committees have equal representation from both partners, and alliance managers from both sides are part of each committee (Wellenreuther et al., 2012). Since start of the alliance, more than 100 project proposals have been received, more than 30 joint projects have started and 20 milestones were achieved in 16 projects. One of these projects will start clinical development in the near future. Based on this experience, Ruth Wellenreuther and Holger Hess-Stumpp identified a number of key success factors: collaboration on equal foot level with close interaction, exchange of expertise and mutual benefit and long-term perspective; the alliances enabling scientists to translate their research into applications; a framework agreement enabling quick setup of joint projects (not requiring single project negotiations); low administrative hurdles with streamlined and short decision processes; low bureaucracy with fixed personnel and consumable rates and flexibility in deployment of funds; incentives for successful application, completion of milestones and appropriate reporting; participation of departments and scientists of DKFZ in financial revenues.

Partnerships involving multiple pharmaceutical companies and multiple academic and other organizations were discussed by Matthias Gottwald (Bayer, Berlin, Germany), based on the example of IMI. IMI is an overarching scheme of the European Union and the European Federation of Pharmaceutical Industry Associations (EFPIA) (Laverty and Goldman, 2014). It has three aims, to make the R&D process in Europe more innovative and efficient, to enhance Europe’s competitiveness and to address key societal challenges. IMI started in 2008 and is currently planned to run through 2024. Over this entire time frame, the European Union and the EFPIA members have pledged more than 5 billion €, with half of this sum coming from each side. The contribution from industry is largely provided “in kind,” i.e., by making manpower, technologies and samples available. In contrast, the contribution from the European Union is provided “in cash” and distributed to the public partners in the consortia. Such public partners include academic institutions, patient organizations and regulatory authorities as well as small-to-medium sized enterprises. Industry defines the overall scope of the projects, and public consortia can apply for participation via competitive calls. Bringing together the complementary expertise of the different stakeholders and experts from across Europe allows to address issues that none of the partners could or would address alone. In the first phase of IMI (2008–2013) with a budget of 2 billion €, more than 7000 researchers representing 845 academic teams, 17 regulatory authorities, 26 patient organizations, 169 small-to-medium enterprises and 480 EFPIA teams have worked or currently are working together in about 60 projects. These projects cover all parts of the R&D value chain from drug discovery up to market access. An example for a project improving the efficiency of the drug discovery process is the EU Lead Factory^1^, in which seven companies have shared compounds from their own libraries to create a big joint library, complemented by specifically synthesized compounds coming from the public consortium partners, and linked to a central screening center which allows to screen novel targets against this highly diverse library. By using this approach several companies have already identified novel compounds for their pipeline which they could not have found in their own libraries. In the preclinical safety project eTOX^2^ 12 pharma companies and several public partners have shared data from their preclinical *in vivo* toxicity studies thereby creating the biggest database in this field. Together with newly developed modeling and simulation approaches this allows a better prediction of potential toxicity linked to novel compounds, allowing to deselect development compounds with a safety risk months earlier than today. The project Electronic Health Records for Clinical Research EHR4CR^3^ has developed standards for electronic health records and an IT infrastructure for clinical trial feasibility studies against patient registered at multiple centers in an anonymized way, thereby offering the potential to avoid protocol amendments at a later stage and to speed up patient recruitment. And the Get Real project^4^ analyzed data used for discussion with HTA agencies to develop a guidance for HTA data collection during late clinical development with the aim to increase the probability that the relevant required data for a successful dialog with the agencies are collected early in the process.

A significant share of the budget (39%) went to projects related to infectious diseases. Other major areas of funding included drug discovery, brain disorders, metabolic disorders, drug safety, stem cells, cancer and data management. IMI projects have so far identified more than 460 new biomarker candidates, developed or standardized more than 50 new animal models, more than 100 new *in vitro* models and more than 100 new *in silico* models. More than 20 new drug targets were identified and more than 25 new tools were established to facilitate drug development. Data generation for a better understanding of diseases on a molecular level and validation of a multitude of biomarkers and other tools was done in more than 65 clinical trials including more than 18,000 patients. Specific modular training programs, e.g., for pharmacoepidemiology and pharmacovigilance (EU2P^5^, Translational Safety Sciences (SafeSciMET^6^), and Medicines Development (PHARMATRAIN)^7^ have been developed, attended already by more than 1000 trainees. On the commercial side, 13 new spin-offs were created, 6 trademarks established, 3 licensing deals made, and 7 projects commercialized. Moreover, 20 patent applications were filed, with 65% of them coming from academia. The second phase of IMI (2014–2020) with an increased joint budget of more than 3 billion €, will focus on processes and tools to bring precision medicines faster to the patients. To integrate the new opportunities resulting from digital health activities, it will be open to non-EFPIA companies such as those related to healthcare IT and medical devices. IMI2 will improve funding for small-to-medium enterprises to incentivize broadest possible participation of experts in the respective areas. The overall success of IMI is also witnessed by the fact that South Korea and Japan are starting similar programs on a smaller scale. Examples of broad, multi-stakeholder PPPs are listed in **Table 1**.

### Investigator-Initiated Studies

A special form of PPPs, initiated by academia, is the so-called investigator-initiated research, in case of clinical research often referred to as investigator-initiated trials or investigator-initiated studies (IIS) (Suvarna, 2012). Aspects of non-clinical investigator-initiated research have been discussed in the previous section and reviewed elsewhere (Modjtahedi et al., 2014; Michel et al., 2015; Michel and Korstanje, 2016) Focusing on clinical IIS, Stefan Schröder and Miriam Bach (Bayer, Berlin, Germany) reported that the investigator is responsible for initiating, managing and financing the study, following applicable laws and regulations. These include the rules of Good Clinical Practice and Good Manufacturing Practice as well as article 2 (e) of the European Directive 2001/20/EC. Thus, the investigator or his/her institution assumes full responsibility of the role of sponsor of the study as defined by Good Clinical Practice guidelines. Of note, funding from a pharmaceutical company for an IIS must not be used for any other purpose. The legal aspects of IIS were additionally covered by Sigrid Achenbach (Bayer, Berlin, Germany). She emphasized that IIS must not be promotional but be of scientific merit and promote legitimate research interests. The role of a company in IIS is to provide scientific advice, financial support and/or study drug. In the framework of an IIS, a pharmaceutical company must not plan or conduct a study, draft the study protocol or publication.

In most cases, IIS are performed following regulatory approval of a new treatment and focus on specific subgroups of patients within the approved indication or on potential new indications. While IIS most often fall into the area of competitive research, an IIS can also be co-funded by more than one company both on competing or different products.

In view of the utility of IIS in obtaining additional knowledge on effects of drugs, several companies have now created dedicated websites that investigators can use to submit their specific proposals. Similarly, academic institutions increasingly see IIS as an important means to perform studies of interest, particularly those not funded by government. As an example, Sein Schmidt [Berlin Institute of Health (BIH), Berlin, Germany] presented the BIH. This research institution has been created in 2015 by the German Research Ministry, the state of Berlin and the Helmholtz Society to integrate experimental and clinical expertise at the Charité, one of the leading hospitals in Europe, and the Max Delbrück Center, an important center in biomedical research. This newly formed institute will focus on the translation of novel findings from research into clinical application and back and thereby also play a major role in IIS initiated and/or performed in Berlin.

An even wider network of investigator-driven research is the European Clinical Research Infrastructure Network (ECRIN^8^) It was created in 2004 to become an organization that could facilitate multinational clinical trials in Europe. ECRIN’s first project (2004–2005) focused on strategy, the second (2006–2008) on tools, and the third (2008–2012) on infrastructure development. By 2013, ECRIN had become what it set out to be: a non-profit organization, based on country membership, supporting mostly academic sponsors and investigators across Europe to overcome the barriers to multinational clinical research, now including 80 sites in 23 countries.

While support of IIS may be attractive for a company, it involves risks. For instance, lack of upfront planning could lead to non-validated data unsuitable for publication. While insufficient budget may not allow completion of an IIS, a too high budget may be seen as undue influence by a company, raising issues of compliance and anti-corruption laws. Other than these legal implications, such issues may also damage the reputation of the academic institution and the supporting company. A frequent discussion point in the determination of an appropriate IIS budget is institutional overhead. While including overhead is possible in principle it must be reasonable, particularly considering that the investigator and not the company had initiated the study.

## Adaptive Approaches

One approach to make drug development more cost-effective is the use of adaptive trial designs, which was discussed by Stefan Hantel (Boehringer Ingelheim, Ingelheim, Germany). After the introduction of the overall idea of adaptive trial design (Bauer and Köhne, 1994), this concept has been discussed controversially. The term ‘adaptive trial design’ is used to describe methodology that allows to change key design features of a clinical study based on (unblinded) observed data within the trial and concomitantly controlling the overall type I error level. In draft guidance issued in February 2010, the FDA has defined adaptive trial design as follows (U.S. Food and Drug Administration, 2010): “For the purposes of this guidance, an adaptive design clinical study is defined as a study that includes a prospectively planned opportunity for modification of one or more specified aspects of the study design and hypotheses based on analysis of data (usually interim data) from subjects in the study.” Thus, adaptive trial design has to be pre-specified in the protocol of the study and should not be added during the conduct of the study. ‘Adaptive trial design’ typically does not refer to classic protocol amendments covering minor aspects of a clinical trial such as modification or clarification of in- and exclusion criteria. Adaptive trial design shall also not get confused with adaptive licensing (see below). When considering adaptive design, one must be aware of risks for trial integrity. Thus, results of an interim analysis can potentially jeopardize trial integrity and introduce bias if investigator and/or patients become aware of interim results, which can influence their decisions. It can also affect trial integrity if sponsor or steering committee modifies trial characteristics. On statistical grounds, an interim analysis can have an impact on the type I error due to multiple testing. While stopping a trial for futility (Michel et al., 2013) has likely no impact on the error level of the trial, in can influence the validity of the results of the entire program.

Sample size re-assessment can be based on re-assessment of variability or of overall event rates in event-driven studies and is usually regarded as lacking impact on type I error as long as blinding is maintained. Sample size re-assessment of unblinded data is a common adaptation and provides more detailed information concerning treatment effect. This can be handled statistically by the promising zone approach (Mehta and Pocock, 2011). In this approach, the initial sample size calculation is based on the assumed treatment effect and variability and the targeted power of the study. The observed treatment effect will be calculated in a pre-planned interim analysis, which allows calculation of conditional power based on the observed treatment effect. If the power is lower than planned (i.e., the observed treatment effect smaller than expected), sample size may be increased if the interim results are in a ‘promising zone’. If the treatment effect is much smaller than expected, as expected or higher, the trial continues as planned without adaptation. The EXAMINE trial of cardiovascular outcomes in diabetic patients treated with the dipeptidyl peptidase IV inhibitor alogliptin is an example of adapted sample sizes (White et al., 2013). The trial assessed first non-inferiority of alogliptin based on the margins 1.8 and 1.3 followed by the option to test superiority. The interim analysis for non-inferiority for the 1.3 margin was scheduled after 550 observed events. As pre-specified in the study protocol, sample size could be increased to 1300 events if the results were ‘promising’ in the interim analysis. In this case, the interim analysis did not suggest that the sample size should be increased. The final analysis was based on 621 events in 5380 randomized patients with a hazard ratio of 0.96 with an upper limit of the confidence interval of 1.16 (<1.3). The strength of the adaptive design of the EXAMINE trial is illustrated by the fact that a similar trial without adaptive design also showed non-inferiority without showing superiority; however, that trial was planned to observe 1040 events in 16,500 patients, i.e., required much higher patient numbers leading to a longer trial duration and greater trial cost (Bhatt and Mehta, 2016). Of note, it is being recommended that details of adaptive trial design should not be communicated to the investigators to avoid potential bias. Rather, investigators should only be informed, that the trial will randomize up to 13,000 patients and that the trial has the option to show superiority.

**FIGURE 3:**
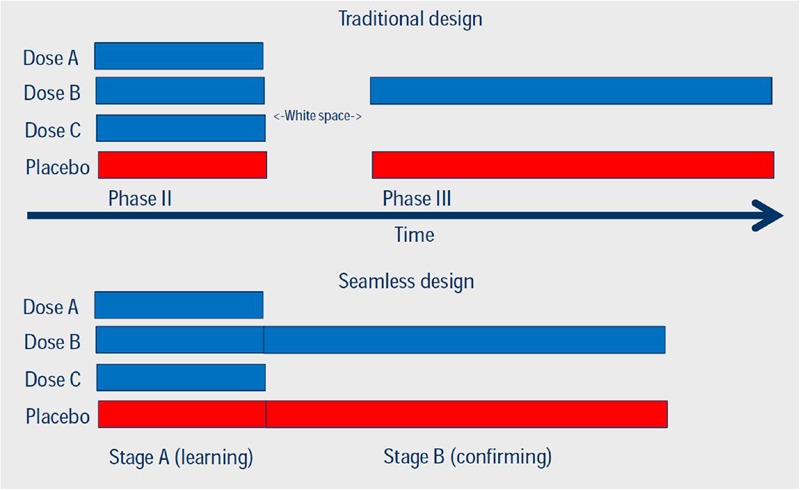
**Comparison of traditional and seamless design of Phases II and III studies.** Reproduced with permission of originator (Dr. S. Hantel, Boehringer Ingelheim Pharma GmbH & Co KG).

Another example how adaptive trial design can speed up clinical development is the idea of seamless phase II/III design. This approach combines two stages, which traditionally are represented by distinct trials. In the traditional approach, the phase II study focuses on selecting the dose for the confirmatory/pivotal trials, whereas the phase III studies confirm efficacy/effectiveness and safety of the selected dose. In a seamless design, the decision is made during the course of the trial and implemented before the study is concluded, preferably while the recruitment is still ongoing. Both stages can be analyzed separately, which results in less concern for bias in the estimators. Several methods exist to combine data obtained in both stages (**Figure 3**). Seamless phase II/III design requires careful considerations, especially if the two stages are using different primary endpoints, e.g., a biomarker vs. survival, a narrower vs. a broader population and/or different regions where the patients are recruited. Such seamless design only is appropriate if the combination of results obtained in both stages make sense from a medical perspective.

Biomarker-based enrichment is another type of adaptive trial design. This uses an (established) predictive biomarker to enrich the patient population (Wang et al., 2007). Such trials start with recruiting biomarker-positive and negative patients. Treatment effects in the biomarker-negative patients are assessed at interim analysis; if treatment in the control group is larger than with the test drug and the difference exceeds a futility boundary, the accrual of biomarker-negative patients will be terminated, whereas biomarker-positive patients continue to be enrolled until the planned sample size has been achieved. The key advantage of this design is a larger power than in a non-adaptive design. A key limitation is that the number of accrued biomarker-positive patients is larger than in a study testing positive patients only and, accordingly, may lead to a longer trial duration.

In conclusion, adaptive trial design can lead contribute to efficiency in drug development by shortening timelines. However, three key conditions must be met to do so:

– The overall type I error should be strongly controlled.
– Opportunities for adaptation should be defined prospectively.
– The time point of the interim analysis has to be planned prospectively.

Overall, it is mandatory that investigators fully understand these methods according to the FDA guideline. They also need to realize that adaptive designs require more intensive planning and discussion within the sponsor and between sponsor and regulatory authorities. Interim analyses should be performed independent of the trial team and results have to be kept confidential. Extensive simulations prior to start of the trial should inform the characteristics of the design. Moreover, the confirmatory character of a study may become questionable if the design involves too much adaptation. Under no condition should adaptive design be used as an excuse for sloppy planning.

Based on the potential benefits of adaptive design, Solange Rohou (Astra Zeneca, Paris, France) reported on the IMI project **A**ccelerated **D**evelopment of **A**ppropriate

**P**atient **T**herapies: A **S**ustainable, **M**ulti-stakeholder **A**pproach from **R**esearch to **T**reatment-outcomes (ADAPT-SMART^9^), which kicked off in June 2015. This project brings together various pharmaceutical companies with regulatory authorities (e.g., European Medicines Agency), health technology assessment bodies (e.g., NICE, HAS), patient organizations (e.g., Eurordis, European Patient Forum) and, as observers, payers. It is aimed at facilitating and accelerating the availability of Medicines Adaptive Pathways to Patients (MAPPs) and thereby fostering access to beneficial treatments for the right patient groups at the earliest appropriate time in the product life-span in a sustainable fashion. The project has to identify tools and methods that could help progress MAPPs while analyzing gaps and barriers to the use of MAPPs and ultimately make corresponding recommendations. For instance, a workshop on MAPPs selection criteria has been organized in February 2016 at the request of the Dutch regulatory authority as part of the Dutch presidency of the EU Council, witnessing the high level of attention this project is being given. There will also be specific discussions involving Health Technology Assessment bodies and payers on managed entry agreements of MAPPs products with potentially great health benefits but considerable uncertainty that would need to be adequately addressed. A key challenge when securing a targeted MAPPs marketing authorization will be to ascertain that commitments to generate additional data are met and that the product will not be used off-label.

Solange Rohou addressed three misconceptions on the use of adaptive pathways. Firstly, adaptive approaches will not provide regulatory approval based on lower standards of evidence. This generates a quid pro quo for drug developers. Rather, evidence will increase over time where multiple decision points change timing of patient access. Earlier market access will be gained in exchange for continued monitoring and label changes based on such monitoring. Second, adaptive approaches will not necessarily be faster and cheaper than traditional pathways. Timing will only be faster to a first decision but overall may increase to enlarge the population to benefit from the product, or if ongoing monitoring does not leverage payer and clinical systems. This implies that the entire development program through indication roll-out and surveillance is agreed upon early. Third, patients will not gain unfettered early access. Rather patients would likely participate in tracking, registries and observational studies with associated informed consent. The ADAPT-SMART consortium will be successful if it identifies all opportunities and challenges to active patient participation, while developing a clear roadmap to operate MAPPs in the near future. It will require self-challenge and the courage to experiment with the understanding that there are many different views among stakeholders including payers; there is the shared intention to do the best to find solutions to make the concept viable and resolve open questions together.

## Big Data

The term “big data” has been coined in the mid-1990s by John Mashey, a computer scientist at Silicon Graphics, but remains ill-defined (Flockhart et al., 2016) as explained by Wolfgang Renz (Columbia University, New York, NY, USA and McGill University, Montreal, Canada). Most often “big data” is used as an umbrella term to describe large amounts of data in a general sense, which by virtue of their volume may allow combination and analysis to uncover unexpected patterns and hidden information. With respect to healthcare, these data mostly come from physicians and hospitals, health insurance companies, and pharmaceutical and medical device R&D; however, they may also involve patient behavior and sentiment as well as population and public health data. In an even broader sense, they may also include data from genomic sources and large scale phenotyping efforts (Syn et al., 2016).

There is a plethora of opportunities stemming from the use of big data, and turning them into practical uses is just beginning. For instance, pharmaceutical and other healthcare companies can apply big data analysis to aggregate information from previous clinical trials to identify potential problems or adverse events. Big data may also allow analysis of clinical data in real time to incorporate insight gleaned from behavior of similar drugs under development, something of potential interest not only to pharmaceutical companies but also to regulatory authorities. Analysis of real-world data such as insurance claims can be seen as giving a voice to customers in evaluating drug or device effectiveness. Another example is medical information coming from life-style products such as wearables (Mombers et al., 2016). Thus, big data offer opportunities along the entire value change from exploring epidemiology, drug target identification to clinical development (Schultz, 2013). Big data may also influence healthcare decision making by reducing waste and inefficiencies and indicate appropriate standardization of care, thereby allowing cost savings. They also facilitate a more customer-focused approach to communication (Mills and Ghatty, 2012).

Non-medical sources such as data collected by Google or Facebook may also allow analysis to provide individual health-related information. A case making the news in early 2012 may illustrate this (Hill, 2012). The US store chain Target had identified sets of customer behavior that in aggregation have a high chance of detecting pregnancy and even forecasting a likely delivery date, which can be used in sending targeted advertisement to corresponding customers. This became public when the father of a high-school student protested that his daughter received pregnancy-related advertisements from Target. Unknown to him but apparently rightly predicted by Target use of consumer data, his teenage daughter was indeed pregnant. In biomedical terms, one may conclude that Target apparently had identified an unconventional but novel biomarker of pregnancy.

The use of big data provides the opportunity to enable experimentation, create new knowledge and transparency; this may allow to customize and target products and services, improve decision making and promote innovation. Turning this potential into practice faces challenges. While some sources of big data are well structured, many are highly heterogeneous and very dispersed even if they were generated within a major pharmaceutical company (Harris, 2013). Thus, strategies for data management and data segregation are still being developed, as is the capacity to generate and communicate knowledge from big data. One can only imagine the difficulty of physician scientists acting as referees for a manuscript submitted to a major biomedical journal in judging the scientific quality of data, if the algorithms they are derived from are intellectually and mathematically highly complex and far away from daily medical practice. Therefore, a major challenge in the use and analysis of big data is to design a process how to present all of the information in a manner accessible and understandable to users. Alliances between classic data analysis companies such as SAS and major pharmaceutical companies such as GSK are a logical answer to these challenges (Munro, 2013). Other challenges include the lack of a clear regulatory and legal framework for the use of big data in drug development. This includes the privacy rule in the Health Insurance Portability and Accountability act in the US or data protection and privacy legislation. On technical grounds, protecting data in centralized stores is another potential challenge. The IMI has recently started to address these challenges via a so called Big Data for Better Outcomes (BD4BO) program, in which data from various sources like real life data, Electronic Health Records, clinical trials, patient registries and others will be made available and novel approaches for the analysis of these heterogeneous data will be developed. If successful, this offers the opportunity to support better treatment decisions and a better guidance for a more targeted development. Of note, proof of efficacy and safety of new medications for obtaining marketing authorization is likely to remain a domain of randomized clinical trials. Big data may help to address questions of healthcare efficiency but factors such as channeling and utilization bias will need to be considered in the interpretation of such data.

### Informed Consent and Privacy

Perhaps the most important societal challenge in the use of big data is building public trust among citizens and patients that big data will be used in an appropriate manner and privacy will be maintained (Larson, 2013). Moreover, the use of big data and biobanks of human specimens raises fundamental questions on the type and validity of informed consent. Ulla Ohlms (PATH Biobank, Munich Germany) presented the patient view on informed consent, specifically with regard to biobanking. She highlighted that modern patients no longer obtain knowledge on their condition and available treatments from the yellow press but increasingly use the internet for information gathering. They tend to have faith in molecular biology research and expect tailored therapy based on molecular information. This infuses the patient-physician relationship with more shared decision making. In this spirit, the PATH Foundation has been established in 2002 by members of the breast cancer union “mamazone e.V.” as a patient-driven biobank for breast cancer. It is a joint venture of physicians, scientists and patients to provide a resource for breast cancer research. It aims to support cancer research in academia and industry by providing biomaterials linked to clinical data including follow-up information. It is a non-profit organization but charges users of the specimens a cost recovery fee. Presently, it has stored about 5000 samples each from tumor and normal tissue and about 7500 serum samples from more than 8000 patients (Waldmann et al., 2014). Recent research has shown that consent procedures vary widely, even within a country, and this variability includes factors such as addressing right of withdrawal, genetic analysis and transborder use (Hirschberg et al., 2013). The PATH foundation has taken several steps to ensure full informed consent of donors. This includes comprehensive written information and a clear consent form with the option of additional information via internet, e-mail and phone. The consent material and the PATH foundation process for sample use have been reviewed by an independent ethical committee, a medical lawyer and the state privacy officer and are regularly controlled by PATH’s board of trustees. Key issues are a consent for use in cancer research in a broad definition, lack of time limits on storage, transfer of property rights to the biobank, the right to withdraw at any time and data protection by pseudonymization. To ensure patient engagement and full transparency, donors receive a yearly newsletter informing them about news about the biobank, latest information from cancer conferences as well as projects involving PATH samples. Moreover, projects using PATH samples and resulting publications are listed on the internet^10^.

Based on this experience, Ulla Ohlms discussed which issues in informed consent forms are important from a patient point of view. One aspect is how samples will be used and what kind of analysis is planned. Particularly when applied to whole genome analysis, becoming increasingly frequent in cancer research (Syn et al., 2016), the question arises whether and how incidental findings shall be communicated to the donor (Bishop et al., 2016). Other relevant issues include a definition which third parties may use the samples and whether they may be used in countries having privacy protection laws different from those where the donor resides. As some of the issues around use of samples, for instance the increase in whole genome sequencing, may not have been foreseen at the time of obtaining a sample, the concept of dynamic consent has developed (Kaye et al., 2015). This concept connects patients and researchers to enable more efficient patient recontact but remains to be widely implemented.

Stefan Brink (Data Protection Officer Rhineland-Palatinate, Mainz, Germany) discussed the limits of informed consent from a legal point of view. As privacy and data protection laws differ considerably between legislations, he focused on the situation in Germany. This has largely been shaped by a 1983 landmark ruling of the German Federal Constitutional Court. It ruled that the German constitution “warrants the capacity of the individual to determine in principle the disclosure and use of his/her personal data.” Implementing the ruling of the Constitutional Court, § 3 of the German Federal Data Protection act defines “Personal data means any information concerning the personal or material circumstances of an identified or identifiable individual (the data subject).” The legal definition challenges the use of big data, particularly related to “identifiable.” The association and integration of various sources of information may make an individual identifiable in ways that had not been anticipated when that law was enacted. Said §3 further defines “Special categories of personal data means information on a person’s racial or ethnic origin, political opinions, religious or philosophical convictions, union membership, health or sex life.” It further states “Rendering anonymous means the modification of personal data so that the information concerning personal or material circumstances can no longer or only with a disproportionate amount of time, expense and labor be attributed to an identified or identifiable individual.” When applying these definitions, anonymization becomes almost impossible in big data. Therefore, use of data collected under German legislation in big data analysis may be violating German law. However, there is ongoing discussion whether related to medical including genomic data the public good may outweigh the benefits of keeping information private.

As discussed by Jill Nina Theuring (Bayer, Berlin, Germany), data protection laws cover the healthcare sector but are general and have not been designed specifically for this sector. Progress in the collection and use of big data is driven by rapid technological advances in this field, and legislation cannot easily keep up with the speed of the technological advances, not to mention predict what those advances will be by the time the legislation becomes effective. This legal framework is further complicated by differences between global areas, within the EU and even between local regulatory agencies in Germany. The new European general data protection regulation, which was adopted in April 2016 and will enter into force on May 25th 2018, is likely to include opening clauses permitting deviations in member states. Taken together this situation creates considerable legal uncertainty in the healthcare sector. Such uncertainty leads to various individual approaches of regulatory authorities. Dealing with this heterogeneity is costly and time-consuming for those involved in biomedical research and drug development, limits pan-European and global projects, and may put institutions located in some EU member states at a competitive disadvantage compared to those in others.

The trend toward more personalized/stratified medicine increases the need to obtain and process clinical data and those obtained from human biospecimen. Preclinical research activities including genome-wide association studies, whole genome sequencing and genetic tests in conjunction with biomarkers also contribute data. This trend is further promoted by the requirements of regulatory authorities to provide data supporting the use of biomarkers and companion diagnostics in patient-tailored drugs. Data privacy laws are relevant for all kinds of pharmaceutical activities from early research to real-life evidence; academic research in this regard is similarly affected. Therefore, an aligned understanding of legal requirements between pharmaceutical companies, academics, contract research organizations, regulatory authorities, ethical committees, data protection officers and patients is crucial.

One step to face this challenge has been taken via so-called Coordination and Support Action for the IMI2 BD4BO program, to be launched in January 2017. This will drive the health outcomes strategy of the BD4BO program, integrate knowledge and disseminate findings, design approaches to ensure sustainability of projects in the program, ensure consistency and quality across projects and to bring and share expertise across all diseases and themes. One key element of the work will be the analysis of the data privacy environment in Europe and beyond and the joint work on a harmonization of Informed Consent across stakeholders and countries.

## Conclusion

The rising cost of drug development (Herper, 2012; Scannell et al., 2012) combining with increasing pressure on drug pricing (Scannell, 2015) challenge current business models of the pharmaceutical industry. Public–private partnerships, adaptive designs and use of big data offer opportunities for greater efficiency in drug development. Only the future will tell which of these approaches has helped most. For the time being, we feel that all of them are promising candidates to make drug R&D more efficient; their best admix is likely to be a project-specific question. However, these opportunities can only be leveraged if the inherent limitations of each of these are understood. For all of these areas it can be concluded that it is no longer pharmaceutical industry in isolation that will develop new drugs. Rather we propose that early dialog between pharmaceutical companies, academic investigators, regulatory authorities, health technology assessment bodies, payers and, most importantly, patients will increasingly shape the way new drugs are developed to address existing medical needs.

## Author Contributions

OY has contributed to the concept development of the manuscript, drafted part of the text, has approved the final version and takes full responsibility for the manuscript. MG and PS has participated in the program development of the underlying symposium, contributed to the concept development of the manuscript, drafted part of the text, has approved the final version and takes full responsibility for the manuscript. MM has participated in the program development of the underlying symposium, contributed to the concept development of the manuscript, drafted part of the text, coordinated the overall writing, has approved the final version and takes full responsibility for the manuscript.

## Conflict of Interest Statement

OY is a present employee of Boehringer Ingelheim. MG is an employee of Bayer Pharma AG. PS is an employee of Icon. MM is a past employee of Boehringer Ingelheim.

## Footnotes

1 www.europeanleadfactory.eu

2 www.etoxproject.eu

3 www.ehr4cr.eu

4 www.imi-getreal.eu

5 www.eu2p.org

6 www.safescimet.eu

7 www.pharmatrain.eu

8 www.ecrin.org

9 www.adaptsmart.eu

10 www.path-biobank.org
